# Effects of Eutrophication and Different Water Levels on Overwintering of *Eichhornia crassipes* at the Northern Margin of Its Distribution in China

**DOI:** 10.3389/fpls.2019.01261

**Published:** 2019-10-04

**Authors:** Haihao Yu, Xianru Dong, Dan Yu, Chunhua Liu, Shufeng Fan

**Affiliations:** The National Field Station of Freshwater Ecosystem of Liangzi Lake, College of Ecology, Wuhan University, Wuhan, China

**Keywords:** eutrophication, water drawdown, invasion, overwintering, *Eichhornia crassipes*

## Abstract

When exotic species are introduced into new areas, establishment is a vital step in their invasion process. Therefore, overwintering strategies determine whether an exotic species from low latitudes can successfully invade middle- and high-latitude areas. In this study, we investigated the effects of nutrient and water drawdown on overwintering in an exotic aquatic plant from the tropical zone, *Eichhornia crassipes*, at the northern margin of its distribution in China. The population density, size of individuals, and the size and nitrogen concentration of overwintering organs (stem base) of *E. crassipes* that grew in high-nutrition water were greater than those that grew in low-nutrient water before winter. The overwinter survival rate of *E. crassipes* was significantly affected by the water level and nutrient. The thick and dense floating mat of *E. crassipes* can increase the temperature of water bodies; therefore, the overwinter survival rate of *E. crassipes* was higher in constant-water-level and high-nutrient treatment. In contrast, due to the loss of heat preservation provided by the floating mats and the low nitrogen concentration in the stem base, all individuals of *E. crassipes* died in constant-water-level and low-nutrient treatment. In the water-drawdown treatments, the stem base of *E. crassipes* was directly exposed to low-temperature air; therefore, the overwinter survival rate of *E. crassipes* was lower. Our results reveal that eutrophication can not only improve the competitiveness of *E. crassipes* but can also improve the survival rate of overwintering plants in temperate regions. Our study also suggests that removing nutrients from the water and regulating the water level can limit the invasion of *E. crassipes* in temperate and subtropical regions.

## Introduction

When exotic species are introduced to new ranges, only a few species can establish populations and become invasive. Propagule pressure and the self-sustaining ability of species in adverse environments mainly influenced the establishment (Williamson and Fitter, 1996a; Williamson and Fitter, 1996b). In high-altitude and high-latitude regions, overwintering abilities and strategies are critical for the establishment, distribution, and spread of exotic species, especially for species that originated from habitats in warmer regions. Studies of the overwintering ability of species and factors that affect overwintering allow us to clarify the mechanisms of invasion and better control them.

Overwintering is the process by which some organisms pass through or wait out the winter season. During this period, organisms experience numerous kinds of abiotic (cold or sub-zero temperatures, frost, frost heave, ice, snow, low precipitation, drought) and biotic (limited food supplies, low-temperature fungi, and bacteria) stresses, making normal activity or even survival difficult or near impossible (Bertrand and Castonguay, 2003). Temperature is a limiting factor for survival, growth, and reproduction in plants and many animals (Woodward, 1987; Charnov and Gillooly, 2003). Low-temperature and freezing events in winter result in the invasion failure of many introduced exotic species or restrict the distribution ranges of species that have successfully invaded (Owens et al., 2004; Walther et al., 2009; MacIsaac et al., 2016). Although climate warming causes exotic species to invade regions within which they could not survive before, extends their temporal and spatial distribution, and increases their performance (Walther et al., 2002; Hellmann et al., 2008; Walther et al., 2009; Chown et al., 2012; Concilio et al., 2013; Sorte et al., 2013), the ranges of some exotic species may also be reduced (Merow et al., 2017). In some ranges, precipitation was also low in winter, which leads to a decline in the water levels of lakes and rivers. Water level is an important factor affecting the growth and reproduction of aquatic plants in freshwater ecosystems (Chambers and Kalff, 1985; Ishii and Kadono, 2004; Deegan et al., 2006; Smith and Brock, 2007; Xiao et al., 2007; Xie et al., 2008). Moreover, water is usually considered to be a temperature buffer to prevent aquatic plants from direct damage by freezing in winter (You et al., 2013). Therefore, water level changes can also influence the overwintering of exotic species.

Nutrients are also another important abiotic factor affecting species growth, propagation, or colonization, and as one of the results of global changes that is driven by human activities and rapid economic growth, eutrophication has become increasing common and severe in water systems (Ryther and Dunstan, 1971; Smith et al., 1999). Previous studies found that the relative growth rates, reproductive rates, photosynthetic rates, leaf nitrogen contents, and photosynthetic nitrogen-use efficiencies of aquatic exotic plants increase more intensively with increasing nutrient availability than those of native species (Xie et al., 2010; Fan et al., 2013). However, few studies have examined the effects of eutrophication on the overwintering of exotic plants. With the intensification of global change, water level fluctuation (e.g., floods and droughts) will become more intense and frequent, and eutrophication will also be more severe and widespread in freshwater ecosystems (Arnell and Reynard, 1996; Allen et al., 2001; Schindler, 2006). Studies the effects of water level changes and eutrophication on the overwintering of exotic aquatic plants can help to predict and manage exotic aquatic plants.

One of the world’s most prevalent invasive aquatic plants, *Eichhornia crassipes* (water hyacinth), is a free-floating and mat-forming aquatic plant that originates in tropical South America. Now, *E. crassipes* has invaded over 50 countries on five continents (Villamagna and Murphy, 2010). It occurs in various freshwater ecosystems (estuaries, rivers, lakes, ponds, reservoirs, and canals); forms thick, extensive mats; and causes severe ecological and socio-economic changes in where it has invaded (Mitchell, 1985; Center, 1994). *E. crassipes* reproduces both sexually and asexually. In invaded regions, it increases in population size mainly through vegetative reproduction, forming new ramets from axillary buds on stolons produced through the elongation of internodes (Center and Spencer, 1981). Sexual reproduction rarely occurs, owing to the lack of suitable pollinators and appropriate sites for germination and seedling establishment in invaded regions (Barrett, 1980). Temperature and water nutrient levels are key factors that affect the invasion of *E. crassipes* (Wilson et al., 2005). The optimal growth temperatures for water hyacinth are 28–30°C; growth stops if the water temperature falls below 10°C or rises above 40°C (François, 1969; Gopal, 1987), and the plant dies when it experiences prolonged cold temperatures below 5°C (Gopal, 1987; Owens and Madsen, 1995). The edge of the distribution of *E. crassipes* occurs where the mean temperature in January is 1°C, the mean annual temperature is 13°C, and the average lowest temperature during the year is −3°C (Ueki et al., 1976). The distribution of *E. crassipes* is considered to be limited to tropical or subtropical regions because it cannot overwinter in environments with extreme cold temperatures or ice cover (Aurand, 1982; Tyndall, 1982; Madsen et al., 1993). However, some researchers have predicted that its distribution may expand into higher latitudes as temperatures rise (Rodríguez-Gallego et al., 2004; Hellmann et al., 2008; Rahel and Olden, 2008). The growth and reproduction of *E. crassipes* are closely related to the nutrient level of water bodies (Reddy et al., 1989; Reddy et al., 1990; Xie et al., 2004). Some studies found that a high-nutrient supply can improve the photosynthetic capacity, resource-use efficiency, and competitiveness of *E. crassipes* (Ripley et al., 2006; Fan et al., 2013). In addition, both the depth of the water and changes in water level are important in the growth of *E. crassipes* (Téllez et al., 2008). For example, the results of study of Oki and Ueki (1984) indicated the leaf area and growth rate of *E. crassipes* in shallow water were higher than those in deep water, while more roots were found in the latter. Moreover, some researchers have found fluctuations in water level promoted the invasion of *E. crassipes* (Freidel et al., 1978; Téllez et al., 2008).

*Eichhornia crassipes* was introduced into China as an ornamental plant in the early 1900s and is now widely distributed in 17 provinces or cities and causes severe damage in more than 10 provinces. In tropical China, water hyacinth can grow all year round. In subtropical regions, the plant dies back in winter and sprouts new plants from axillary buds on the stem base the following year (Gao, 2005). In the middle and lower reaches of the Yangtze River, the overwintering survival rate of water hyacinth is very low (Gao, 2005). However, we knew little about the overwintering mechanisms of water hyacinth (You et al., 2013; Liu et al., 2016). In order to test whether eutrophication and water level changes can facilitate the overwintering of *E. crassipes* as well as climate warming and found the reasons, we investigated the effects of nutrients and water drawdown on the overwintering of *E. crassipes*, at the northern margin of its distribution in China. We attempted to address the following questions: (i) Do high nutrient levels affect the performance of *E. crassipes*? (ii) Do different treatments lead to different overwintering temperatures of *E. crassipes*? (iii) Can high nutrient levels or water cover increase the overwintering survival rate of *E. crassipes*?

## Materials and Methods

### Study Site

The experiment was conducted at The National Field Station of Freshwater Ecosystem of Liangzi Lake, Wuhan University, China (30°05′−30°18′N, 114°21′–114°39′E). Liangzi Lake is a shallow lake with an area of 304.3 km^2^ in the central reaches of the Yangtze River basin. The climate of this area is a typical subtropical climate. The average temperature in winter ranges from 3 to 7°C, which is the critical temperature for the overwintering of *E. crassipes*; therefore, Liangzi Lake is located at the northern margin of the *E. crassipes* distribution in China (Li and Xie, 2002).

### Experimental Design

**Figure 1:**
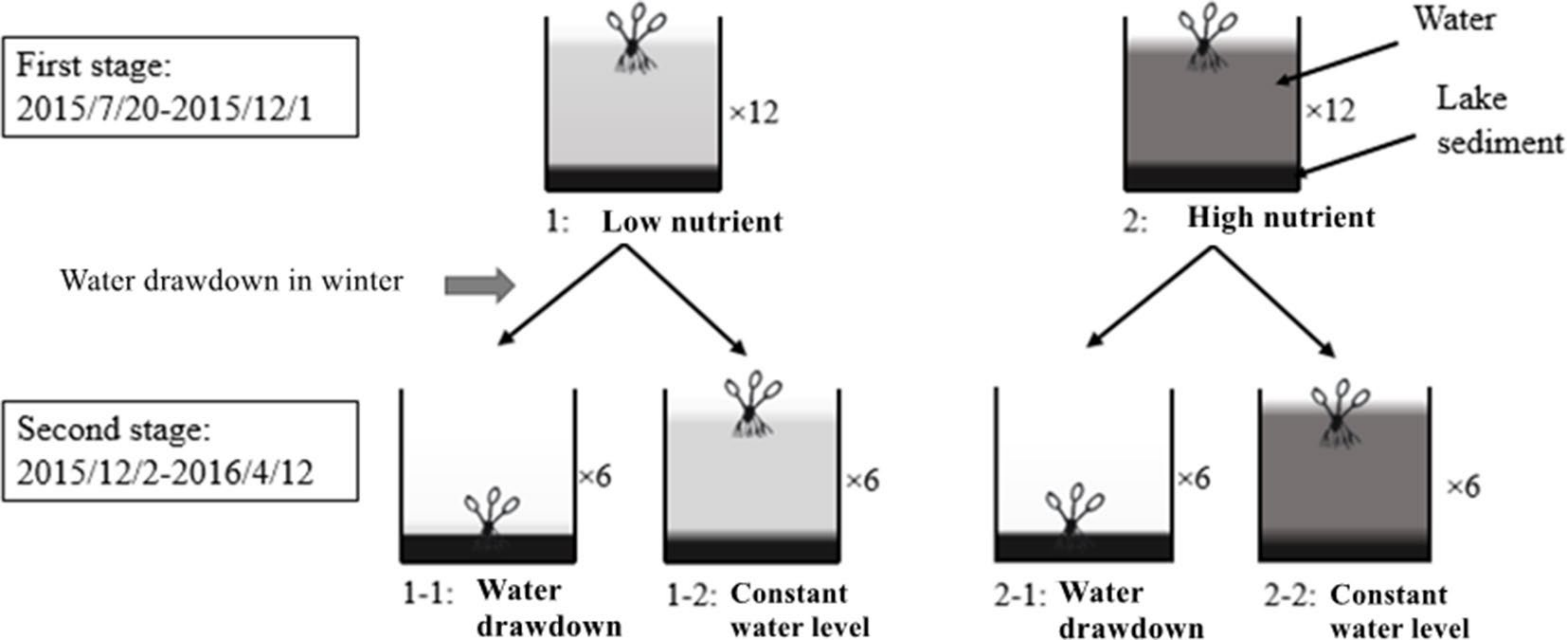
Diagram of the experimental design (the number following the “×” indicates the number of replicates. One plant in diagram represents 10).

In early July 2015, *E. crassipes* plants were collected from the bay of Liangzi Lake and cultivated in a 4 × 4 × 4-m concrete pool. Fifteen days later, 240 healthy plants of similar size (mean fresh biomass 63.56 ± 5 g, mean height 14.28 ± 3 cm, mean leaf number 12 ± 3) were transferred to 24 concrete pools (length: 2 m, width: 2 m, depth: 1.5 m) filled with approximately 10 cm of lake sediment (from Liangzi Lake, N:P = 2.35:0.014 mg/L) and 120 cm of water (from Liangzi Lake, N:P = 0.6:0.05 mg/L); each pool was planted with 10 plants. The whole experiment lasted about 35 weeks, from July 20, 2015, to April 12, 2016, and was divided into two stages (**Figure 1**). During the first stage (growing stage, from the beginning of the experiment to December 1, 2015), the pools were divided into two treatments: low nutrients and high nutrients, and each nutrient level had 12 replicates (n = 12). During the second stage (overwintering stage, from December 2, 2015, to the end of the experiment), a water level factor was added to half of the pools in those two treatments until the experiment finished; there were therefore four treatments during this stage: 1-1: low nutrients and water drawdown; 1-2: low nutrients and a constant water level; 2-1: high nutrients and water drawdown; and 2-2: high nutrients and a constant water level. Each treatment had six replicates (**Figure 1**).

During the growing stage, in the high-nutrient treatment, we increased the total nitrogen (TN) and total phosphorus (TP) concentrations to 20 mg/L N and 1.0 mg/L P by adding KH_2_PO_4_, NH_4_NO_3_, and 10% Hoagland solutions to the lake water. In the low-nutrient treatment, only 10% Hoagland solution was added to the lake water. To maintain a relatively constant concentration of the culture solution, an appropriate amount of nutrients was added into relevant pools every half month after the nutrient level was measured. At the end of the growing stage, the temperature dropped with the onset of the yellowing of leaves, causing the plants to stop growing. Then, the experiment entered the second stage (overwintering stage). In the water-drawdown treatments, the water was drained (no water over sediment) by pump. In constant water level treatments, the water level was kept in line with that of the first stage. And the water levels of each treatment after rain were maintained by pumping. No nitrogen phosphorus fertilizer was added to each treatment during the second stages, because the plants have stopped growing. To study question (ii), do different treatments lead to different overwintering temperatures of *E. crassipes*? the temperature of the microenvironment in which the plants were located was monitored at 30-min intervals with four automatic thermometers during the overwintering stage. These thermometers were placed in four pools selected randomly from the four different treatments. In the water-drawdown treatments, the thermometer probes were placed closed to the stem base of the water hyacinth. In the treatments with a constant water level, the thermometer probes were placed in the surface layer of water (5 cm from the surface of the water) where the stem base of the water hyacinth was occurred.

### Data Collection

To answer question (i), do high nutrient levels affect the performance of *E. crassipes*? we counted the plants in each pool and harvested three plants randomly from each pool to measure the traits before winter at the end of the growing stage. The length and diameter of the stem base were measured with a Vernier caliper. The biomass of the plants and the stem base were obtained after drying plants in an oven at 70°C for 72 h to a constant weight. The soluble sugar and starch concentrations of the stem base were measured by the sulfuric acid anthrone colorimetric method described by Hansen and Moller (1975). The nitrogen and carbon concentrations of the stem base were analyzed by a FLASH 2000 Organic Elemental Analyzer (Thermo Fisher Scientific Inc., USA). 

To answer question (iii), can high nutrient levels or water cover increase the overwintering survival rate of *E. crassipes*? we counted the number of surviving plants (with the original plant and its new ramets counted as one plant) approximately every 10 days from early March 2016 (when some plants began to regrow) to April 12, 2016 (when the weather became warm enough to ensure that all plants with survival potential survived successfully). In addition, we identified the plants with new green leaves as those that had survived. The final survival rate was calculated as the number of plants that had survived divided by the number of plants at the end of the growing stage.

### Data Analysis

To answer question (i), do high nutrient levels affect the performance of *E. crassipes*? One-way ANOVA was performed to examine the effects of nutrients on plant number, plant biomass, stem base biomass, stem base length, stem base diameter, and the soluble sugar concentration, starch concentration, carbon concentration, and nitrogen concentration in the stem base. To test question (ii), do different treatments lead to different overwintering temperatures of *E. crassipes*? the differences in the mean temperatures of the microenvironment in which the stem bases of the plants were located among the different treatments were also tested by one-way ANOVA. To answer question (iii), can high nutrient levels or water cover increase the overwintering survival rate of *E. crassipes*? number of plants after the winter was tested with a general linear model (Poisson distribution), which indicated that both the nutrient level and water level significantly impacted the number of surviving plants. Then, two-way ANOVA was used to test the impacts of nutrient level and water level in winter and their interaction effect on the survival rate and number of surviving plants after winter. The survival rate, length and weight of the stem base, number of plants before winter, and soluble sugar concentration of the stem base were transformed using the SQRT function to ensure the homogeneity of the variance or a normal distribution of the residuals before the analysis. All data were analyzed with SPSS 19.0 software (SPSS, Chicago, IL, USA).

## Results

### Growth Traits During the Growing Stage

**Figure 2:**
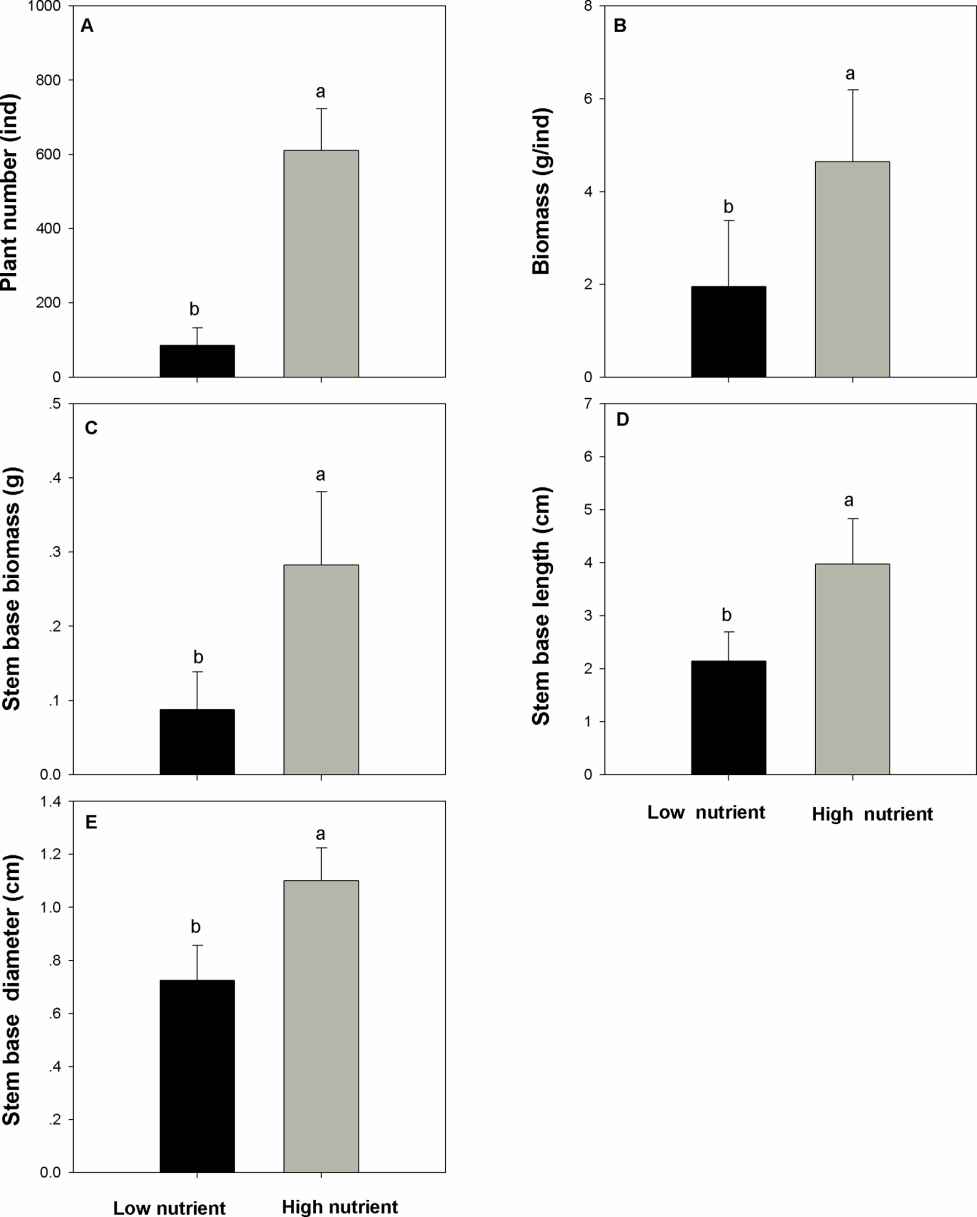
Number **(A)**, biomass **(B)**, stem base biomass **(C)**, stem base length **(D)**, and stem base diameter **(E)** (mean ± SD) of *Eichhornia crassipes* under different nutrient treatments before winter. Significant differences among the treatments are indicated by different letters at P < 0.05.

**Figure 3:**
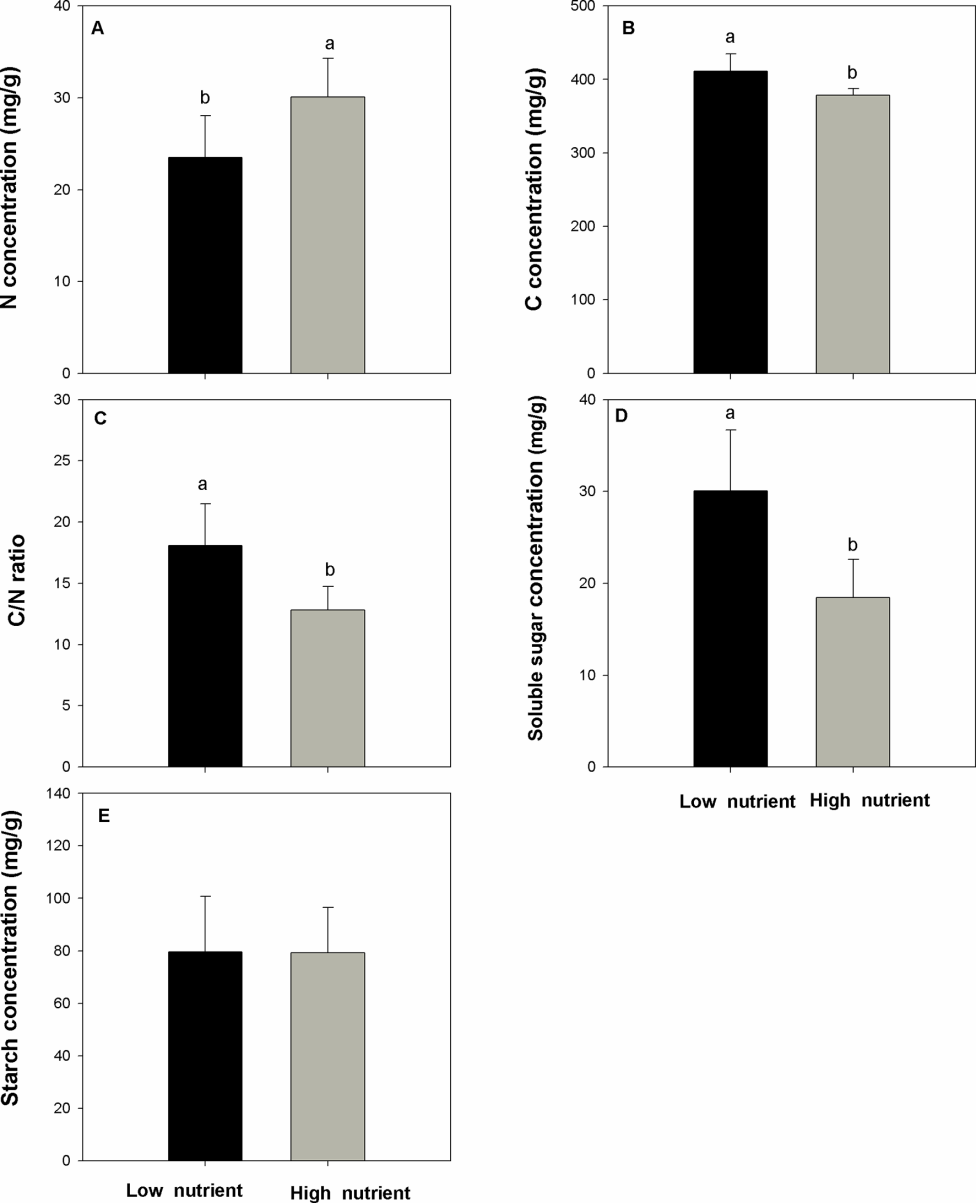
N concentration **(A)**, C concentration **(B)**, C/N ratio **(C)**, soluble sugar concentration **(D)**, and starch concentration **(E)** (mean ± SD) in the stem base of *Eichhornia crassipes* under different nutrient treatments before winter. Significant differences among the treatments are indicated by different letters at P < 0.05.

Nutrient addition significantly and positively affects the populations of *E. crassipes* and the performance of the overwintering stem base (question i). The number of *E. crassipes* plants in the high-nutrient treatments was higher than those in the low-nutrient treatments (**Figure 2A**, F=219.227, P < 0.001). Biomass of single plants in the high-nutrient treatments was also higher than those in the low-nutrient treatments (**Figure 2B**, F = 19.625, P < 0.001). The population of *E. crassipes* in the high-nutrient treatments covered the whole surface of the water and formed dense, interlocking mats; the mean density reached 152 ind/m^2^. However, in the low-nutrient treatments, weaker plants were scattered on the surface of the water, and the mean density of *E. crassipes* was 21.23 ind/m^2^. The biomass, length, and diameter of the stem base in the high-nutrient treatments were all higher than those in the low-nutrient treatments (**Figures 2C–E**; F_biomass_ = 36.722, F_length = _38.491, F_diameter = _51.417; P_biomass_ < 0.001, P_length_ < 0.001, P_diameter_ < 0.001). The high nutrient level also significantly increased the N concentration in the stem base (**Figure 3A**, F = 13.562, P < 0.01). But the C concentration, C/N ratio, and soluble sugar concentration in the low-nutrient treatments were higher than those in the high-nutrient treatments (**Figures 3B–D**; F_C = _19.353, F_C/N = _21.358, F _sugar_ = 26.571; P_C_ < 0.001, P_C/N_ < 0.001, P_ sugar <_0.001). There was no difference in the starch concentration between the high- and low-nutrient treatments (**Figure 3E**, F = 0.002, P > 0.05).

### Microenvironment Temperature and Survival Traits During the Overwintering Stage

In winter, the temperatures of the microenvironments in which the stem bases of the plants located were different (question ii). The mean temperature and minimum temperature of the water around plants in the constant-water-level treatments were higher than the mean temperature and minimum temperature of the air near the plants in the water-drawdown treatments (**Table 1**). In contrast, the maximum temperatures were higher in the latter treatments. In the constant-water-level treatments, the mean temperature and minimum temperature in the high-nutrient treatment were higher than those in the low-nutrient treatment (**Table 1**). In water-drawdown treatments, the mean temperature and minimum temperature under high nutrient level were lower than those under low nutrient level (**Table 1**).

**Table 1:** Microenvironment temperature in winter. Significant differences among the treatments are indicated by different letters at *P* < 0.05.

Treatments	Temperature of air near plants (ºC)			Temperature of water around plants (ºC)		
	Mean	Max.	Min.	Mean	Max.	Min.
**Low nutrient + water drawdown**	6.41c	16.1	−2.8			
**Low nutrient + constant water level**				8.09b	14.1	−1.2
**High nutrient + water drawdown**	6.33d	17.1	−4.0			
**High nutrient + constant water level**				8.10a	11.1	5.2

**Figure 4:**
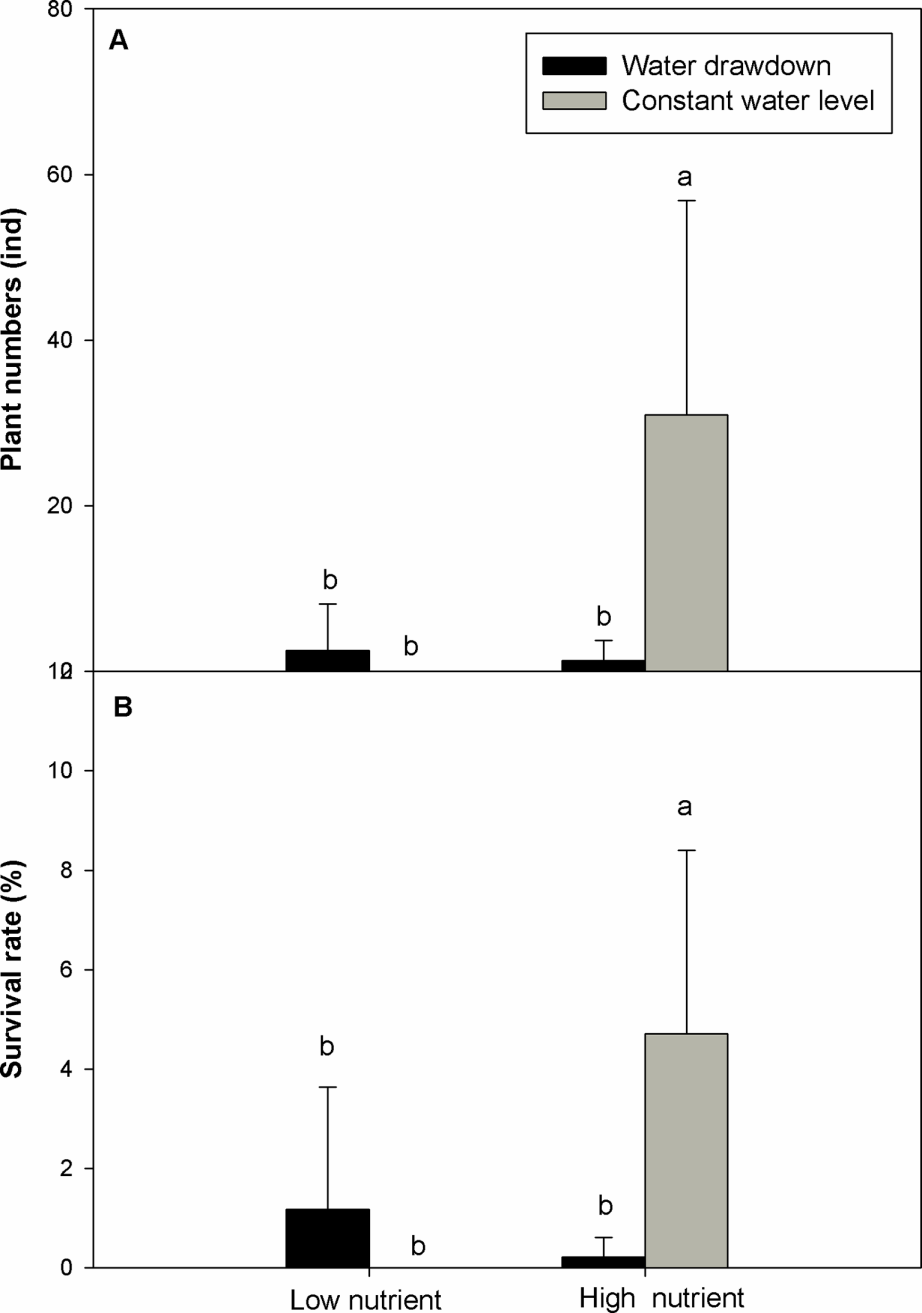
Plant number **(A)** and survival rate **(B)** (mean ± SD) of *Eichhornia crassipes* in different treatments after winter. Significant differences among the treatments are indicated by different letters at P < 0.05.

The final survival rate and total number of plants after overwintering were significantly affected by affected by nutrient level, water level, and their interaction (**Table 2**) (question iii). The high-nutrient treatments increased the survival rate and number of *E. crassipes* plants after overwintering (**Table 2**, **Figures 4A, B**). Under the high-nutrient conditions, water drawdown in winter significantly decreased the survival rate and total number of *E. crassipes* plants after overwintering (**Figures 4A, B**). *E. crassipes* in the high-nutrient and constant-water-level treatment exhibited the highest survival rate (4.71 ± 3.69%) and number of surviving plants (31 ± 25.84), which were markedly much higher than those in the other three treatments, and there were no significant differences in survival rate among the other three treatments. It is worth noting that all the plants died in the low-nutrient and constant-water-level treatment (**Figures 4A, B**).

**Table 2:** Effects of nutrient and water level in winter on survival rate and number of surviving plants of *Eichhornia crassipes* after winter (two-way ANOVA).

Trait	Nutrients		Water level		Nutrients × water level	
	F	P	F	P	F	P
Survival rate	7.365	0.013*	3.567	0.074	14.280	0.001***
Number of surviving plants	13.560	0.001***	7.327	0.014*	15.242	0.001***

*P < 0.05; ***P < 0.001

## Discussion

In our study, both nutrients in the water and water level affected the survival rate of *E. crassipes*. *E. crassipes* originated in the tropics, and the low temperature in winter limits its distribution in introduced regions. Previous studies have shown that survival, growth, and clonal integration in *E. crassipes* are limited by low temperatures (Li et al., 1995, Wilson et al., 2005); therefore, increasing the temperature in winter can increase the survival rate of *E. crassipes* (You et al., 2013; Liu et al., 2016). In China, the Yangtze River basin occurs at the northern margin (32°N) of the distribution of *E. crassipes*. Although *E. crassipes* can grow and blossom in summer at higher latitudes, it cannot naturally survive in winter in China (Li and Xie, 2002). Similarly, *E. crassipes* also cannot overwinter successfully in the Laurentian Great Lakes, although it can grow and produce seed in the summer in this area (MacIsaac et al., 2016). The different treatments caused the stem bases of *E. crassipes* (overwintering organ) was exposed to different media, resulting them being subjected to different temperatures in winter. In our study, the covering of the stem bases with water can prevent direct damage from freezing and improve the overwintering temperature of the propagule. Therefore, the survival rate of* E. crassipes* in constant-water-level treatments was significantly higher. Previous studies also showed that water cover or sediment burial of stem bases facilitated the overwintering of *E. crassipes* (Owens and Madsen, 1995; You et al., 2013).

Similar to water cover, the litter layer can also buffer the cold temperatures and protected organisms under litter from freezing damage during winter and can therefore improve the survival rate of the organisms (Facelli and Pickett, 1991). Litter may also protect seedlings from being killed by frost in early spring (Heady, 1956). Previous studies have found that litter cover can improve the survival rates of animals and plants (Watt, 1970; Lahiri et al., 2015; Miura et al., 2017). In high-nutrient environments, *E. crassipes* plants are taller and larger and can form dense, interlocking mats. In winter, the large number of withered leaves can form a thick litter layer in high-nutrient treatments. In contrast, in the low-nutrient treatments, the litter layer was absent because of the low population density and the small and sparse leaves of *E. crassipes*. The water temperature was higher in the high-nutrient treatments than in the low-nutrient treatments because of the protection of the litter layer in winter. Moreover, the litter cover can also prevent the axillary buds away from the freezing damage.

The *E. crassipes* in the high-nutrient environment also developed a high-quality overwintering organ. In our study, the biomass, length, and diameter of the stem base in the high-nutrient treatments were all higher than those in the low-nutrient treatments. Meanwhile, the N concentration of the stem base was also higher in high-nutrient treatments than in the low-nutrient treatments. More storage leads to more protective substances to survive stressful environments. Plants with more vegetative storage proteins have been shown to increase in response to short days and low temperatures, improving their winter survival rates (Bertrand and Castonguay, 2003; Avice et al., 2003). Biomass and stem base size can also affect overwintering in *E. crassipes*. You et al. (2013) found that the overwintering survival rate of *E. crassipes* with large stem bases was much higher than that of plants with small stem bases. Although the starch concentrations of the stem base were similar in the high- and low-nutrient environments, the stem base in the high-nutrient environment still stored more starch, as the size of the stem base was larger. More carbohydrate reserves that make large stem bases are beneficial to the regrowth of new plants in the spring. Therefore, our study suggests that high levels of nutrients can improve the overwintering survival rate of *E. crassipes* in two ways: through the production of a thicker litter layer and the storage of more protective substances stored in the stem base.

The high nutrient level not only improved the overwintering survival rate of *E. crassipes* but also increased the performance of *E. crassipes*. In our experiments, the population density of *E. crassipes* in the eutrophic water increased to more than six times than that in the low-nutrient water. The individual biomass in the eutrophic water was 2.4 times that in the low-nutrient water. Our previous study found that, only at a high nutrient level, *E. crassipes* had a higher resource-use efficiency than the confamilial native aquatic plant *Monochoria vaginalis* (Fan et al., 2013). Zhao et al. (2006) also found that eutrophication further boosts the competitive advantages of water hyacinth over native plants and thus facilitates the invasion of this weed into water bodies. During the past 30 years in China, rapid urbanization, gross domestic product (GDP) increases, vast population growth, and living standard improvements have all produced domestic and industrial wastewater. Moreover, due to insufficient sewage treatment capacities, some of this wastewater is discharged, untreated, directly into rivers and lakes (Shao et al., 2006; Yang and Pang, 2006; Le et al., 2010), which causes organic pollution and eutrophication in many water bodies and the replacement of grass-dominated ecosystems with algae-dominated ecosystems (Jin et al., 2005). Seventy-three percent of the major lakes in China have undergone severe eutrophication, and the area of eutrophication amounts to 11,632 km^2^ (Li, 2006). Therefore, our results suggest that *E. crassipes* will spread to a wider area and cause worse effects under the background of intensified eutrophication and climate warming.

Many techniques have been used to control and eliminate the ecological and socio-economic impacts of *E. crassipes* (Villamagna and Murphy, 2010). *Neochetina eichhorniae* and *Neochetina bruchi* are two commonly used weevil species from the plant’s native range (Deloach and Cordo, 1976; Julien and Griffiths, 1999; Sosa et al., 2007). *N. eichhorniae* was introduced into China to control *E. crassipes* in 1995 (Ding et al., 2001). However, the population of *N. eichhorniae* was limited due to low temperatures in the Yangtze River basin. Our study indicated that eliminating eutrophication and regulating the water level can control or even eradicate *E. crassipes* in the Yangtze River basin. The overwintering survival rate of *E. crassipes* in the low-nutrient treatment was less than 5%, even all plants died in the low-nutrient and constant-water-level treatment. Musil and Breen (1977) also considered broad-scale nutrient reduction plans as the most sustainable solution for controlling *E. crassipes* outside of its native range. Even in water bodies with high nutrient levels, the overwintering survival rate of *E. crassipes* can be reduced by the water-drawdown treatment. Previous studies also found that drawdown can be used to manage aquatic vegetation (Nichols, 1975; Cooke, 1980).

In conclusion, our study found that high levels of nutrient not only increased the performance of *E. crassipes* but also improved the overwintering survival rate of *E. crassipes* by producing a thicker litter layer and more protective substances stored in the stem base in at the middle and lower reaches of the Yangtze River basin, which suggests that *E. crassipes* can invade higher latitudes under the trend of climate warming and water eutrophication, whereas exposing the stem base of *E. crassipes* to air of lower temperature by lowering the water level, which can reduce the survival rate of *E. crassipes* in winter. In addition, it also indicated that eliminating eutrophication and regulating the water level can control *E. crassipes* effectively in temperate regions and some subtropical regions.

## Author Contributions

DY, SF, and CL designed the research and executed the research project. HY and XD collected the field data. SF and XD analyzed data. XD and HY wrote the paper.

## Funding

This study was supported by the National Natural Science Foundation of China (31500295 and 31170339), the Major Science and Technology Program for Water Pollution Control and Treatment (2015ZX07503-005), and the Special Foundation of National Science and Technology Basic Research (2013FY112300).

## Conflict of Interest

The authors declare that the research was conducted in the absence of any commercial or financial relationships that could be construed as a potential conflict of interest. 

The handling editor is currently organizing a Research Topic with one of the authors CL, and confirms the absence of any other collaboration.
